# Average and time-specific maternal prenatal inflammatory biomarkers and the risk of labor epidural associated fever

**DOI:** 10.1371/journal.pone.0222958

**Published:** 2019-11-05

**Authors:** Dominique Y. Arce, Andrea Bellavia, David E. Cantonwine, Olivia J. Napoli, John D. Meeker, Tamarra James-Todd, Thomas F. McElrath, Lawrence C. Tsen

**Affiliations:** 1 Department of Anesthesiology, Perioperative and Pain Medicine, Division of Obstetric Anesthesia, Brigham and Women’s Hospital, Harvard Medical School, Boston, Massachusetts, United States of America; 2 Department of Environmental Health, Harvard T.H. Chan School of Public Health, Boston, Massachusetts, United States of America; 3 Department of Obstetrics and Gynecology, Division of Maternal Fetal Medicine, Brigham and Women’s Hospital, Harvard Medical School, Boston, Massachusetts, United States of America; 4 Lake Erie College of Osteopathic Medicine, Erie, Pennsylvania, United States of America; 5 Department of Environmental Health Sciences, University of Michigan School of Public Health, Ann Arbor, Michigan, United States of America; Poissy-Saint Germain Hospital/Versailles Saint Quentin University, FRANCE

## Abstract

**Background:**

The use of labor epidural analgesia has been associated with intrapartum fever, known as labor epidural associated fever (LEAF). LEAF is most commonly non-infectious in origin and associated with elevated inflammatory cytokines.

**Methods:**

The LIFECODES pregnancy cohort was designed to prospectively collect data to evaluate the association of maternal inflammatory biomarkers with preterm birth in women who delivered between 2007 and 2008 at Brigham and Women’s Hospital. Our secondary analysis of the data from the cohort identified 182 women for whom inflammatory biomarkers (i.e. interleukin-10, interleukin-1β, interleukin-6, tumor necrosis factor-α and C-reactive protein) collected longitudinally over four prenatal visits was available. Maternal temperature and other clinical variables were abstracted from medical records. The primary outcome, the presence of LEAF, was defined as oral temperature ≥ 38°C (≥100.4°F) after epidural analgesia initiation. Multivariable logistic regression estimated the association between inflammatory biomarker concentrations and the odds of developing an intrapartum fever after adjusting for a number of potential confounders.

**Results:**

Women who developed LEAF were more likely to have a longer duration of epidural analgesia, whereas women who did not develop LEAF were more likely to have induced labor and positive or unknown Group B *Streptococcus* colonization status. However, no differences were seen by nulliparity, mode of delivery, white blood cell count at admission, baseline temperature, length of rupture of membranes and number of cervical exams performed during labor. Unadjusted and multivariable logistic regression models did not provide evidence for or exclude an association between individual maternal inflammatory biomarkers and the odds of developing LEAF, regardless of visit time-period.

**Conclusion:**

The predictive value of maternal inflammatory biomarkers measured during early- and mid-pregnancy for the risk of developing LEAF cannot be excluded.

## Introduction

Utilized by more than 70% of women undergoing childbirth in the United States, [1] labor epidural analgesia has been associated with intrapartum fever [2,3]. Labor epidural associated fever (LEAF) affects up to one third of deliveries and is thought to account for approximately 90% of intrapartum fever in low risk nulliparous women at term gestation [3,4]. Intrapartum fever, which increases the risk of neonatal encephalopathy, cerebral palsy and unexplained neonatal seizures [5–8], can be caused by non-infectious and infectious etiologies, including intraamniotic infections (IAI) [9].

The etiology of LEAF is most likely a non-infectious inflammatory reaction [10]. Clinical studies supporting this theory include those using methods of fever suppression, including prophylactic intrapartum acetaminophen and dexamethasone administration, and others demonstrating an association of LEAF with baseline and continued elevation of interleukin (IL)-6, a pro-inflammatory cytokine [11–13]. Additionally, a recent animal study used exogenous IL-6 injections to induce fever in near term pregnant rats [14].

Previous studies have examined the association between maternal levels of inflammatory biomarkers and the development of LEAF; however, these studies have largely focused on uncomplicated term pregnancies in nulliparous women at a single time point within labor and delivery. Little is known about whether inflammatory biomarkers at earlier time points in pregnancy could predict the development of LEAF. Thus, we conducted a secondary analysis of prospectively collected samples, examining longitudinal changes—from first to third trimester—in five maternal plasma biomarkers of inflammation among women who subsequently did or did not develop LEAF. These biomarkers include cytokines IL-1β, IL-6, and tumor necrosis factor-α (TNF-α), which are acute phase reactants and known endogenous pyrogens [15], IL-10, an anti-inflammatory cytokine known to inhibit bacterial mediated production of IL-1β and TNF-α [16], and C-reactive protein (CRP), which is produced in response to a rise in IL-6 [15,17]. Evaluating these maternal inflammatory profiles across pregnancy may provide critical information about the development of LEAF for earlier identification of high risk women.

## Materials and methods

### Study population

Women were selected from the ongoing LIFECODES pregnancy cohort at Brigham and Women’s Hospital (Boston, MA), which was initiated in 2006 to study early biomarkers of preterm birth and other pregnancy related disorders. Details about this cohort can be found elsewhere [18,19]. In brief, LIFECODES recruits and collects data, including pregnancy health information, and blood and urine samples, from women at their first and three additional prenatal visits. To be included in LIFECODES, participants had to be ≥ 18 years of age, plan delivery at Brigham and Women’s Hospital (Boston, MA), be <15 weeks gestational age (GA) at baseline and be pregnant with ≤3 fetuses. This study is an analysis of prospectively collected samples. All women included in the study provided written informed consent and the study was approved by the institutional review boards at Brigham and Women’s Hospital (for the overall cohort) and the University of Michigan [18,19]. The manuscript adheres to the STROBE guidelines.

In 2011, a nested case-control study of preterm birth was conducted. From a total of 1,648 women recruited into the cohort at that time, 130 cases of singleton preterm birth and 352 singleton controls were selected for this study; all 482 women had their labor and delivery records abstracted for data on epidural analgesia, maternal temperature, and other clinical variables. These data resulted in 302 women being excluded for at least one of the following criteria: undergoing an elective cesarean delivery (n = 116), having a preexisting autoimmune disorder (n = 26), an inadequate documentation of temperature (i.e. women for whom no temperature data were recorded (n = 10), women who did not have temperature measurement at the time of the epidural catheter placement (n = 13) and at least two additional temperature measurements greater than 2 hours after the epidural placement (n = 68)), or white blood cell (WBC) count ≥17 [20] prior to epidural catheter placement (n = 34), having epidural analgesia < 2 hours (n = 40), or actively taking steroids at admission or during labor and delivery (n = 12). No participants in the study had a fever or documented infection at the time of admission; no participants had a positive intrapartum blood or urine culture. In addition, no participants were excluded solely based on the presence of histological chorioamnionitis from a postpartum placental examination. In total, 180 women were included in the final sample for our analyses.

### Inflammatory biomarker assessment

Maternal plasma specimens were obtained at 4 antepartum visits. Samples were collected with a median GA at visit 1 of 9.7 (range 4.7 to 16.1) weeks, visit 2 of 17.9 (range 14.9 to 21.9) weeks, visit 3 of 26.0 (range 22.9 to 29.3) weeks, and visit 4 of 35.1 (range 33.1 to 38.3) weeks. All specimens were stored at -80°C until analysis [19].

Details about how the inflammatory biomarkers were measured and analyzed are described in detail elsewhere [19]. Briefly, time-point specific maternal plasma samples were analyzed for systemic inflammatory biomarkers at the University of Michigan Cancer Center Immunological Monitoring Core (Ann Arbor, MI, USA). Cytokines were analyzed using Milliplex MAP High Sensitivity Human Cytokine Magnetic Bead Panel (EMD Millipore Corp., St. Charles, MO, USA), individual measures below the limit of detection (LOD) (0.128 pg/mL for all cytokines) reported as numeric values were kept as is and those reported as <0.128 pg/mL were replaced with LOD/√2. C-reactive protein (CRP) was measured using a DuoSet enzyme-linked immunosorbent assay (R&D Systems, Minneapolis, MN, USA) and the lower LOD was 10 pg/mL and upper LOD was 100 pg/mL. As reported previously, the biomarkers were highly detectable in the study cohort, with IL-6 detected in 97.9% of samples and CRP, IL-10, and TNF-α detected in 99.9% of samples. IL-1β, was detected in 78.0% of samples [19].

### Maternal intrapartum fever assessment

The primary outcome for this study was the presence of LEAF, which was defined as an oral temperature of ≥38°C (≥100.4°F) while receiving labor epidural analgesia. Women who had at least one recorded temperature ≥38°C (≥100.4°F) were considered to have LEAF presence. Women with all recorded temperatures <38°C (<100.4°F) were considered to have LEAF absence. All temperature measurements were abstracted from the nursing flowsheet in each participants’ medical record. As noted previously, patients without adequate documentation of intrapartum temperatures were excluded. The temperature measurement at epidural catheter placement was defined as being taken within 2 hours prior or 1 hour after epidural catheter placement. The presence of LEAF was assessed as a dichotomous variable.

### Covariates

All statistical models were adjusted for the following covariates chosen *a priori* based on epidemiologically or clinically identified risk factors for maternal intrapartum fever and variables associated with higher inflammatory biomarker concentrations. These included: maternal age (years), body mass index (BMI) (kg/m^2^) at the first prenatal visit [21], delivery GA (weeks) [20,21], duration of epidural analgesia infusion (hours) [4] and number of cervical exams [22], which were evaluated as continuous covariates; nulliparity [23], dichotomized as having had a previous pregnancy progressing past 20 weeks gestational age or not; race, also dichotomized as being a white versus non-white. Covariates were recorded from data collected in the parent study, as well as in the labor and delivery records.

### Statistical analysis

We first conducted preliminary analyses on demographic and clinical characteristics in the overall populations, as well as stratifying by the presence or absence of LEAF. Continuous covariates were described by calculating means and standard deviation, while categorical covariates were described by calculating the total and relative number of participants in each group. Additionally, we evaluated whether baseline characteristics for women included and excluded from the study were different.

We next evaluated the associations between inflammatory biomarkers and LEAF. First, we compared the unadjusted means of each biomarker concentration, for all time points, between women with and without LEAF. Next, unadjusted as well as multivariable adjusted logistic regression models were used to estimate odds ratios (OR) of LEAF as a function of mean inflammatory biomarker levels, with each of the 5 biomarkers being evaluated in separate statistical models. Due to pronounced skewedness, biomarkers were evaluated in statistical models by using a log-transformation. To relax the assumption of log-linearity in the association between exposures and ORs, this main analysis was replicated by evaluating inflammatory biomarkers as categorical exposures, using quartiles of the distribution. Additionally, we conducted multivariable logistic regression models using log-transformed individual biomarker concentrations, evaluated as categorical exposures, at the fourth time-point in place of the mean.

Finally, several sensitivity analyses were conducted, replicating logistic regression models by additionally including: i) the potential role of labor epidural analgesia duration as a mediator of LEAF by removing it from the multivariable logistic regression model; ii) the role of baseline temperature, by adjusting for this covariate in all statistical models; iii) assessing whether associations between biomarkers and outcomes were non-linear, by flexibly evaluating exposures with restricted cubic splines [24].

All analyses were performed using Stata, version 15 (StataCorp, College Station, Texas). Statistical tests were two-tailed and p-values < 0.05 were considered statistically significant.

## Results

### Participant characteristics

Demographic and clinical data from included and excluded study participants are presented in Table 1. There were no significant differences in the distribution of included versus excluded study participants by race/ethnicity, maternal education or type of health insurance. Women included in the final analysis were slightly younger and more likely to be nulliparous. The included and excluded groups also differed significantly by mode of delivery and Group B *Streptococcus* (GBS) colonization status.

**Table 1:** Baseline characteristics of study participants included vs. excluded in the analysis.

Characteristic	All	Included	Excluded^a^	p-value^b^
N	482	180	302	
Age (y)	32.1 (5.4)	30.8 (5.9)	32.9 (5.0)	0.01
BMI at initial visit (kg/m^2^)	26.3 (6.1)	25.7 (5.4)	26.7 (6.4)	0.10
Race: White	283 (59)	103 (57)	180 (60)	0.61
Maternal education (y)				
• < 12	18 (4)	4 (2)	14 (5)	0.18
• High school/GED equivalent	50 (11)	23 (13)	27 (9)	
• > 12	403 (85)	148 (85)	255 (86)	
Health insurance				
• Self-pay or Medicaid/MassHealth	379 (81)	139 (80)	240 (81)	0.75
• Private insurance/HMO	91 (19)	35 (20)	56 (19)	
Nulliparous	215 (45)	115 (64)	100 (33)	0.01
Smoked during pregnancy	28 (6)	12 (7)	16 (5)	0.53
Use of ART	53 (11)	24 (13)	29 (10)	0.21
GBS status				
• Positive	68 (14)	39 (22)	29 (10)	0.01
• Negative	37 (8)	23 (13)	14 (5)	
• Unknown	377 (78)	118 (65)	259 (85)	
Admission WBC count	11.7 (3.6)	11.1 (2.8)	12.3 (4.1)	0.01
Induction of labor	162 (34)	83 (46)	79 (26)	0.01
Mode of delivery				
• Cesarean delivery	205 (42)	39 (22)	166 (55)	0.01
• Vaginal delivery	253 (53)	128 (71)	125 (42)	
• Assisted vaginal delivery	23 (5)	13 (7)	10 (3)	
Gestational age at delivery (wks)	37.9 (2.9)	38.7 (2.2)	37.4 (3.1)	0.01
Preterm	131 (27)	34 (19)	97 (32)	0.01
Birthweight	3127 (731)	3269 (594)	3043 (791)	0.01
Male infant	214 (44)	83 (46)	131 (44)	0.58
APGAR 5-min < 7	14 (3)	2 (1)	12 (4)	0.07

BMI, body mass index; GED, general educational development; HMO, health maintenance organization; ART, assisted reproductive technology; GBS, Group B *Streptococcus*; WBC, white blood cell; ROM, rupture of membranes. Values are n (%) or mean (SD)

^a^ elective cesarean delivery, missing admission temperature, labor epidural analgesia < 2 hours, white blood cell count >17 prior to epidural placement, preexisting autoimmune disorder, participants actively taking steroids at the time or during the admission for labor and delivery.

^b^ Comparing included vs. excluded only.

The baseline characteristics of all participants (N = 180), composed of those with LEAF presence (N = 35) or absence (N = 145), are presented in Table 2. Overall, the women had a mean age of 30.8 years, a mean BMI of 25.7 kg/m^2^, an unknown GBS colonization status (65%) and a prevalence of preterm birth of 19%. They were predominately white (57%), nulliparous (64%), and had an unassisted vaginal delivery (71%). The women with LEAF presence, versus absence, were less likely to present for an induction of labor (29% vs. 50%) but had a higher mean duration of epidural analgesia (10.1 h vs. 7.5 h). There were no differences between the two groups when comparing parity, mode of delivery, admission WBC count, temperature at the time of epidural catheter placement, duration of rupture of membranes and number of cervical exams performed during labor.

**Table 2:** Baseline characteristics of study participants with epidurals by LEAF status.

Characteristic	All Patients	LEAF (≥ 38°C)	LEAF absence (<38°C)	p-value^*a*^
Sample Size	180 (100)	35 (19)	145 (81)	
Maternal age (y)	30.8 (5.9)	29.5 (4.8)	31.1 (6.1)	0.16
BMI at initial visit (kg/m^2^)	25.7 (5.4)	26.7 (6.0)	25.5 (5.2)	0.23
Race: White	103 (57)	21 (60)	82 (57)	0.71
Nulliparous	115 (64)	23 (66)	92 (63)	0.80
Smoked during pregnancy	12 (7)	1 (3)	11 (8)	0.31
Use of ART	24 (13)	2 (6)	22 (15)	0.14
Gestational age at delivery (wks)	38.7 (2.2)	38.3 (3.2)	38.9 (1.9)	0.16
Induction of labor	83 (46)	10 (29)	73 (50)	0.02
Mode of delivery				
• Cesarean delivery	39 (22)	10 (28)	29 (20)	0.48
• Vaginal delivery	128 (71)	22 (63)	106 (73)	
• Assisted vaginal delivery	13 (7)	3 (9)	10 (7)	
GBS colonization status				
• Positive	39 (22)	5 (14)	34 (23)	0.01
• Negative	23 (13)	15 (43)	8 (6)	
• Unknown	118 (65)	15 (43)	103 (71)	
Antibiotics for GBS prophylaxis	45 (25)	8 (23)	37 (26)	0.74
Admission WBC count	11.1 (2.8)	11.5 (3.1)	11.0 (2.7)	0.40
Duration of ROM (h)	11.3 (19.1)	11.3 (6.8)	11.3 (20.8)	0.99
Number of cervical exams	5.1 (2.4)	5.6 (2.5)	5.0 (2.3)	0.27
Meconium	11 (7)	2 (8)	9 (6)	0.81
Baseline temperature (°C)	36.6 (0.4)	36.7 (0.5)	36.5 (0.4)	0.05
Duration of epidural analgesia (h)	7.9 (4.1)	10.1 (3.8)	7.5 (4.0)	0.01
Magnesium administration	4 (2)	0 (0)	4 (3)	0.37

BMI, body mass index; ART, assisted reproductive technology; GBS, Group B *Streptococcus*; WBC, white blood cell; ROM, rupture of membranes. Values are n (%) or mean (SD)

^a^ Comparing LEAF vs. LEAF absence

### Average pregnancy inflammatory biomarker concentrations

**Fig 1:**
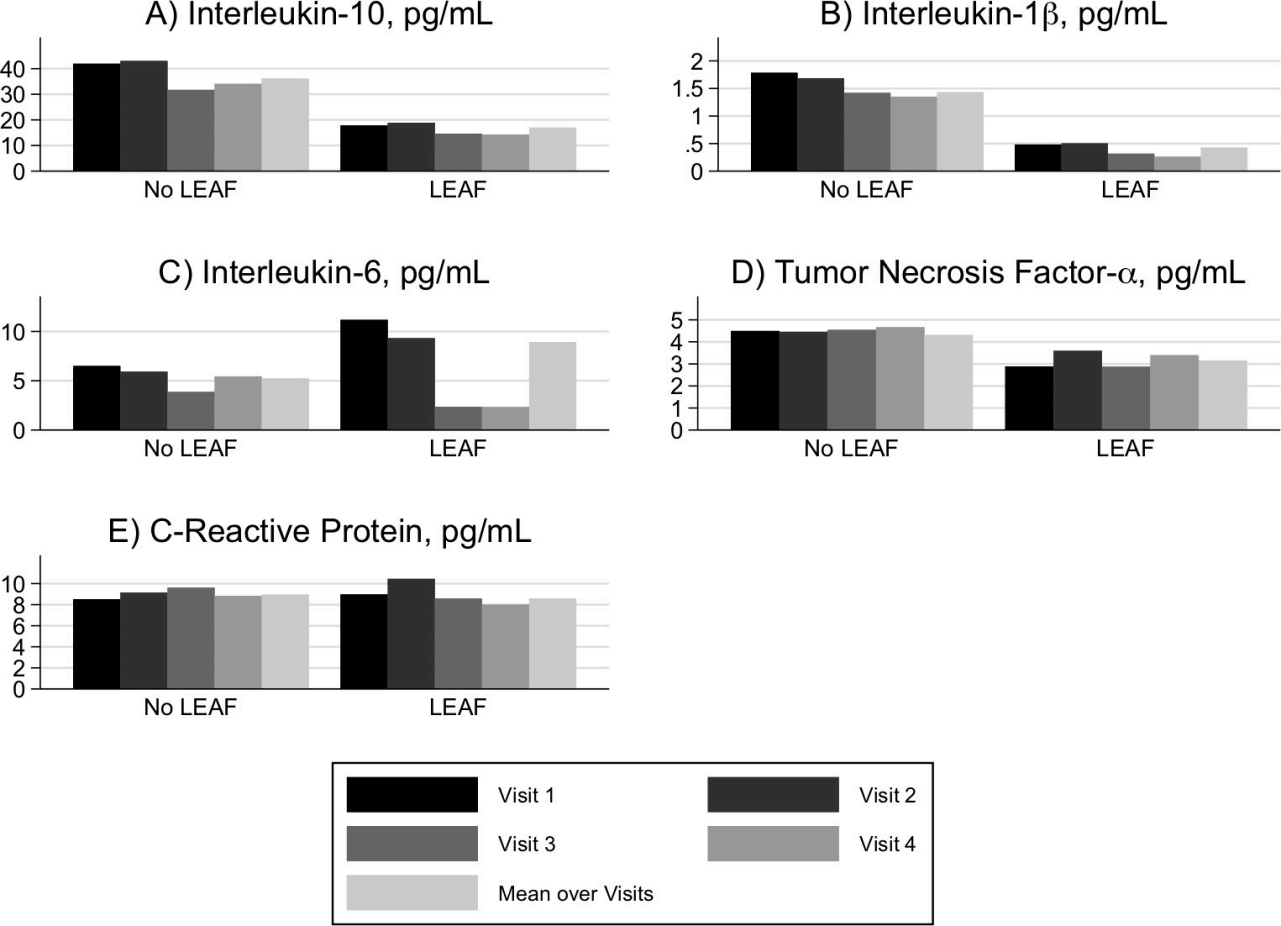
Unadjusted mean concentrations across pregnancy and time-specific average concentrations of the inflammatory biomarkers by LEAF status. LEAF, labor epidural associated fever; IL, interleukin; TNF, tumor necrosis factor, α, alpha; β, beta; CRP, C-reactive protein.

The unadjusted mean concentrations across pregnancy and time-specific average concentrations of the inflammatory biomarkers (IL-10, IL-1β, IL-6, TNF-α, CRP) were compared based on LEAF status (Fig 1). The mean concentrations for IL-10, IL-1β and TNF-α were numerically lower with LEAF presence at each of the four visits individually and collectively. CRP levels were appeared similar in both groups over all time-points, while IL-6 levels were numerically higher with LEAF presence at visits 1 (median 9.7 weeks gestation), 2 (median 17.9 weeks gestation) and mean across pregnancy. However, when unadjusted and multivariable adjusted logistic regression models were performed to test the association between mean concentration of each biomarker and the odds of developing LEAF (Table 3); there was no evidence of an association.

**Table 3:** Association between inflammatory biomarkers averaged across pregnancy and odds of LEAF.

Inflammatory Marker	Unadjusted OR (95% CI)	Adjusted OR (95% CI)^*a*^
IL- 10	0.91 (0.58, 1.43)	0.80 (0.44, 1.45)
IL-1β	0.81 (0.57, 1.14)	0.68 (0.43, 1.08)
IL-6	1.02 (0.74, 1.37)	0.87 (0.55, 1.37)
TNF-α	0.93 (0.53, 1.63)	0.70 (0.35, 1.39)
CRP	0.98 (0.65, 1.48)	0.72 (0.41, 1.27)

LEAF, labor epidural associated fever; IL, interleukin; TNF, tumor necrosis factor, α, alpha; β, beta; CRP, C-reactive protein, OR, odds ratio.

^a^ Multivariable logistic regression adjusted for maternal age, race/ethnicity, nulliparity, BMI at initial visit, gestational age at delivery, duration of epidural analgesia, number of cervical exams.

### Time-Specific pregnancy inflammatory biomarker concentrations

The odds of developing LEAF were investigated for each inflammatory biomarker at visit 4, the time-point closest to the onset of labor and delivery and collectively for all visits, to evaluate the overall inflammatory biomarker concentration throughout pregnancy (Table 4). Only IL-10 showed a positive association between the first (reference) and second quartiles for visit 4, with an OR of 6.09 (95% CI 1.11, 33.45).

**Table 4:** Adjusted associations between average levels of inflammatory biomarkers by quartiles of distribution and odds of LEAF.

	Mean (Visits 1–4) (beta and 95%CI)^a^	Visit 4 (beta and 95%CI)^a^
**IL-10**		
• Q1	1.00 (Reference)	1.00 (Reference)
• Q2	1.24 (0.37, 4.22)	6.09 (1.11, 33.45)
• Q3	0.98 (0.26, 3.75)	2.74 (0.56, 13.41)
• Q4	0.90 (0.25, 3.27)	1.67 (0.27, 10.42)
**IL-1β**		
• Q1	1.00 (Reference)	1.00 (Reference)
• Q2	0.85 (0.26, 2.77)	1.02 (0.25, 4.11)
• Q3	0.89 (0.27, 2.88)	0.42 (0.09, 1.93)
• Q4	0.25 (0.05, 1.31)	0.64 (0.16, 2.65)
**IL-6**		
• Q1	1.00 (Reference)	1.00 (Reference)
• Q2	0.94 (0.28, 3.17)	0.8 (0.19, 3.3)
• Q3	0.22 (0.04, 1.19)	0.34 (0.06, 1.92)
• Q4	0.98 (0.29, 3.3)	1.07 (0.27, 4.21)
**TNF-α**		
• Q1	1.00 (Reference)	1.00 (Reference)
• Q2	2 (0.58, 6.94)	1.11 (0.26, 4.68)
• Q3	1.09 (0.27, 4.32)	1.41 (0.35, 5.65)
• Q4	0.24 (0.04, 1.37)	0.30 (0.05, 1.75)
**CRP**		
• Q1	1.00 (Reference)	1.00 (Reference)
• Q2	1.11 (0.31, 3.91)	1.25 (0.28, 5.53)
• Q3	0.58 (0.14, 2.4)	1.03 (0.23, 4.6)
• Q4	0.61 (0.15, 2.54)	0.54 (0.1, 2.96)

LEAF, labor epidural associated fever, IL, interleukin; TNF, tumor necrosis factor, α, alpha; β, beta; CRP, C-reactive protein.

^a^ Multivariable logistic regression adjusted for maternal age, race/ethnicity, nulliparity, BMI at initial visit, gestational age at delivery, duration of epidural infusion, number of cervical exams.

### Sensitivity analyses

Although the labor epidural analgesia duration was longer in the LEAF group, no evidence was found for an association of labor epidural analgesia duration with any inflammatory biomarkers and LEAF. Furthermore, negligible differences were found when further adjusting for baseline temperature or with restriction of cubic splines.

## Discussion

In our study of pregnant women of mixed parity, only one suggestive association was found between time-specific inflammatory biomarker concentrations (IL-10, IL-1β, IL-6, TNF-α, CRP) collected from the 10^th^ to 35^th^ gestational weeks and the subsequent development of LEAF. The largest association was observed comparing first and second quartiles of IL-10. Overall, the relationship was non-monotonic, indicating that the presence of average concentrations of these inflammatory profiles in early to mid- pregnancy did not confer an increased risk of LEAF. However, given the wide confidence intervals, an association cannot be excluded.

Demographically, women who subsequently developed LEAF differed only in their lower use of labor induction (p = 0.02), lower presence of GBS positive and unknown colonization status (p = 0.01) (but no difference in incidence of GBS antibiotic treatment (p = 0.74)), and longer duration of labor epidural analgesia (p = 0.01) (Table 2).

Our results, which are novel in their correlation to early and mid-pregnancy biomarkers, contrast to those collected on admission for labor and delivery from term (≥ 37 weeks GA), healthy nulliparous women [10,13]. In prior studies, healthy, term, nulliparous women with higher levels of IL-6 on admission to labor and delivery were more likely to develop LEAF than those with lower levels of IL-6 [10,25]. Moreover, the pro-inflammatory cytokines IL-6 and TNF-α, but not IL-1β, have been found to increase with gestation [21]. Such results allowed us to hypothesize that a greater expression of inflammatory mediators could be found in women who develop LEAF, prior to initiation of labor epidural analgesia, possibly at mid-gestation, if not earlier, in pregnancy.

More specifically, we anticipated that among the potent endogenous pyrogens, we would see an increase in IL-1β, which induces fever directly via receptors in the hypothalamus, IL-6 and TNF-α, which are released in response to IL-1β [26], and CRP, which is released in response to IL-6 [17]. In addition, we thought it unlikely that IL-10 levels would increase, which typically occurs in the presence of a bacterial infection[16].

To that end, IL-10 concentrations levels were lower, although not statistically significant, at all visit time-points in women who developed, compared to those who did not develop, LEAF (Fig 1). Mechanistically, this may account for biomarker findings associated with LEAF in prior studies [10, 25]. Lower levels of IL-10, particularly in the setting of an inflammatory stimulus, such as the onset of labor or placement of an epidural catheter [27, 28], can lead to unregulated or elevated levels of TNF-α [27]; TNF-α subsequently augments the production and release of IL-6 [29]. Unexpectedly, IL-1β concentration levels were also consistently lower, yet not statistically significant, at all four time-points in the women with, versus without, LEAF [30]. A potent inducer of IL-6 production, IL-1β is required for IL-6 augmented temperature increases; in the absence of IL-1β [15,31–34], or in knock-out mice without a receptor for IL-1β [35], even the administration of high doses of IL-6 does not result in a febrile response to a noxious stimuli. Additionally, the IL-1 receptor antagonist (ra)/IL-1β ratio, rather than the concentration IL-1β, may be more important in the development of LEAF; fever resulting from pro-inflammatory cytokines is dependent on a reduction in IL-1ra release[36]. Clinically relevant doses of bupivacaine, as used for labor epidural analgesia, have been associated with impaired release of IL-1ra, which mechanistically could explain the temporal relationship between initiation of labor analgesia and the acute onset of fever[36].

As expected, IL-6 concentration levels were consistently low, but not to the level of statistical significance, throughout pregnancy in women who did not develop LEAF, while wide variations were observed in the women who developed LEAF. Lability in IL-6 production may indicate a propensity for an exaggerated response to inflammatory stimuli, which has been observed after epidural placement in women who developed LEAF [25].

Our finding that women who developed, versus did not develop, LEAF had lower prevalence of labor induction and lower presence of GBS positive and unknown colonization status, was contrary to our expectations. Induced labor activity and positive GBS colonization status are associated with increased inflammatory profiles; even inactivated GBS can invoke hyperexpression of maternal and placental IL-1β [37, 38]. However, the administration of oxytocin for inducing labor has been demonstrated to significantly reduce the secretion of TNF-α, IL-6, IL-8, and mitigate the temperature response to endotoxin [39], and therefore may contribute lower associated risk of developing LEAF in women undergoing a labor induction. The GBS colonization status may have had a limited effect, given that antibiotic treatment in women with and without LEAF was not different. Similarly, although the longer duration of labor epidural analgesia is reported as a risk factor in the women with LEAF [23], there were no differences between groups for duration of membrane rupture or number of cervical exams, which are likely more important risk factors for intrapartum fever associated with intraamniotic infection.

Despite our limited findings, the investigation had a number of strengths including a rational hypothesis of gestational events affecting labor outcomes; the LIFECODES parent study found significant associations between increased early and mid-gestation inflammatory biomarkers and preterm delivery [19]. Second, we were able to evaluate and account for a number of potential confounders for LEAF, including nulliparity, age, and race/ethnicity, as well as the number of cervical examinations and the duration of both membrane rupture and epidural analgesia. In keeping with our findings, the number of cervical examinations has recently been observed to not be related to intrapartum fever [40]. Third, we had detailed demographic information and serial temperature measurements in all study participants.

However, we acknowledge several limitations. First, we had limited power due to the small sample size, and we cannot rule out that some of the observed associations may be due to chance. At the same time, the observed associations have a clinically reasonable explanation and there are no comparative studies that utilize repeated measures of pregnancy inflammatory biomarkers for validation. Second, this study utilized clinically available temperature data, which did not occur at precise intervals. To more robustly observe the temperature effect, we excluded those women (26%) who did not have a temperature measurement within 2 hours prior and 1 hour following epidural analgesia initiation. Third, there were some differences between those women who were included and excluded from the analysis. Women who were included were younger, more likely to be nulliparous, preterm, and have their labor induced, and less likely to have a cesarean delivery. Fourth, given the retrospective nature of the study, we were unable to account for all risk factors, including depression and stress, which are known to affect levels of inflammatory biomarkers in pregnant women [41]. As such, the validity in different patient populations may be limited. Fifth, our study was limited to the exploration of biomarkers chosen for examination in the parent study and we were therefore unable to evaluate mechanistic interactions with or the presence of cytokine antagonist.

## Conclusion

In conclusion, our data did not support the hypothesis that higher levels of maternal inflammatory biomarkers measured in early and mid-gestation pregnancy are associated with an increased risk of developing LEAF. However, based on the wide confidence intervals, a relevant association in either direction cannot be excluded. Contrary to previous studies focusing on measures of inflammatory cytokines during labor as an indicator of LEAF, we were not able to confirm that pre-labor measures provide predictive value for identifying women at high-risk of LEAF. Larger, prospective studies are needed to elucidate the association and whether the presence of LEAF may be a consequence of acute inflammatory perturbations immediately prior to or during labor, rather than chronic inflammation that occurs earlier in pregnancy.
